# Persistent painless hemospermia due to metastatic melanoma of the right seminal vesicle

**DOI:** 10.1186/1471-2490-13-43

**Published:** 2013-09-05

**Authors:** Nikolaos Papoutsoglou, Maximilian Burger, Hubertus Riedmiller

**Affiliations:** 1 Department of Urology and Pediatric Urology, Comprehensive Cancer Center Mainfranken, Julius-Maximilian University Medical School, Oberdürrbacher Str. 6, 97080 Würzburg, Germany

**Keywords:** Metastatic melanoma, Hemospermia, Seminal vesicle tumor, Ultrasound, Biopsy

## Abstract

**Background:**

Metastatic melanoma of the seminal vesicles is a very rare clinical entity and has been reported only once until today in a patient suffering from concomitant HIV infection 12 years ago.

**Case presentation:**

We report a case of persistent, painless hemospermia in a young Caucasian caused by metastatic malignant melanoma of the right seminal vesicle. The diagnosis was established by magnetic resonance imaging and transrectal ultrasound-guided biopsy. In the subsequent diagnostic workup the primary location of the tumor remained unknown but concomitant pulmonary, hepatic and supraclavicular lymph node metastases have been detected. Despite immediate chemotherapy initiation the patient finally succumbed to his progressive disease six months later.

**Conclusions:**

Malignant melanoma should be considered as a rare differential diagnosis of hemospermia after common causes have been ruled out.

## Background

Metastatic melanoma of the seminal vesicles is extremely rare. In this manuscript we report a case of persistent hemospermia in a young Caucasian caused by metastatic malignant melanoma of the right seminal vesicle. The first case of metastatic melanoma of the seminal vesicles was reported 12 years ago in a patient with concomitant HIV infection and brain metastases. Pelvic magnetic resonance and ultrasound-guided biopsy were both used as diagnostic tools. Although pulmonary, hepatic and supraclavicular lymph node metastases have been detected the primary location of the melanoma remained unidentified. In spite of active treatment the disease remained progressive and 6 months after the initiation the patient deceased refusing further treatment. Even though metastatic melanomas of the seminal vesicles are extremely rare, hemospermia should be considered as a symptom of this disease after common causes have been ruled out.

## Case presentation

**Figure 1:**
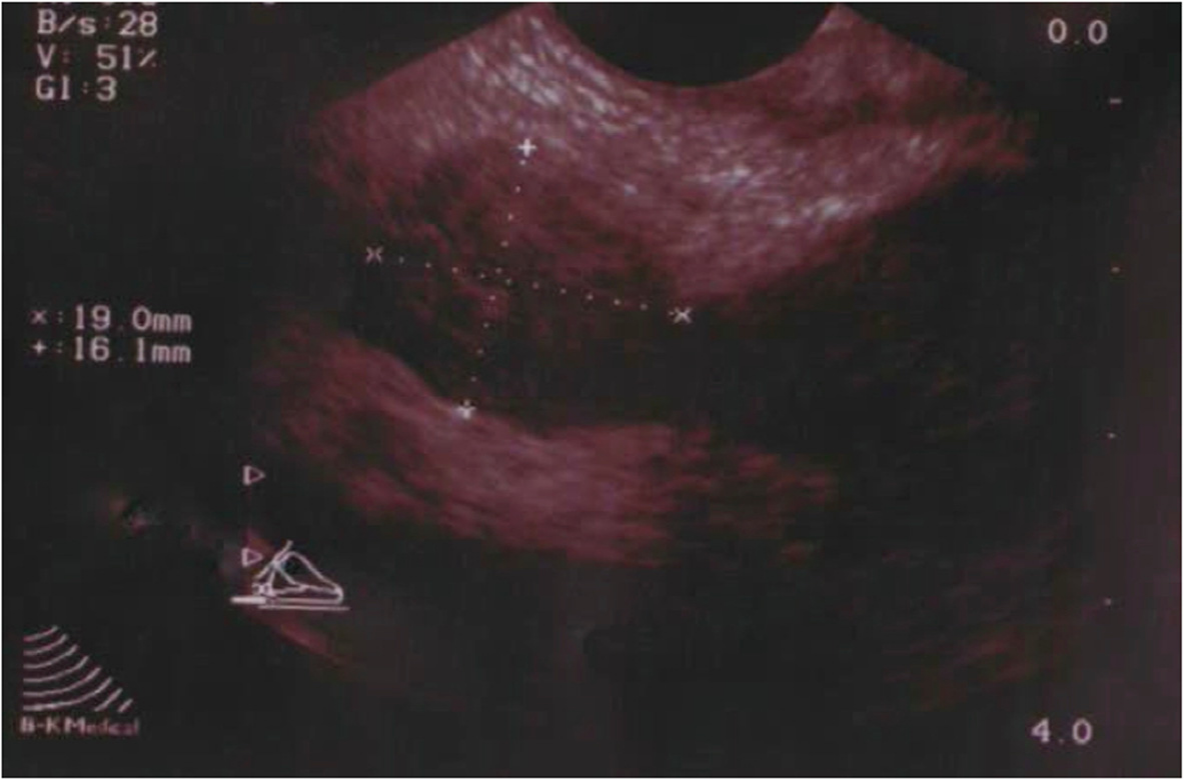
Ultrasound of the right seminal vesicle mass.

**Figure 2:**
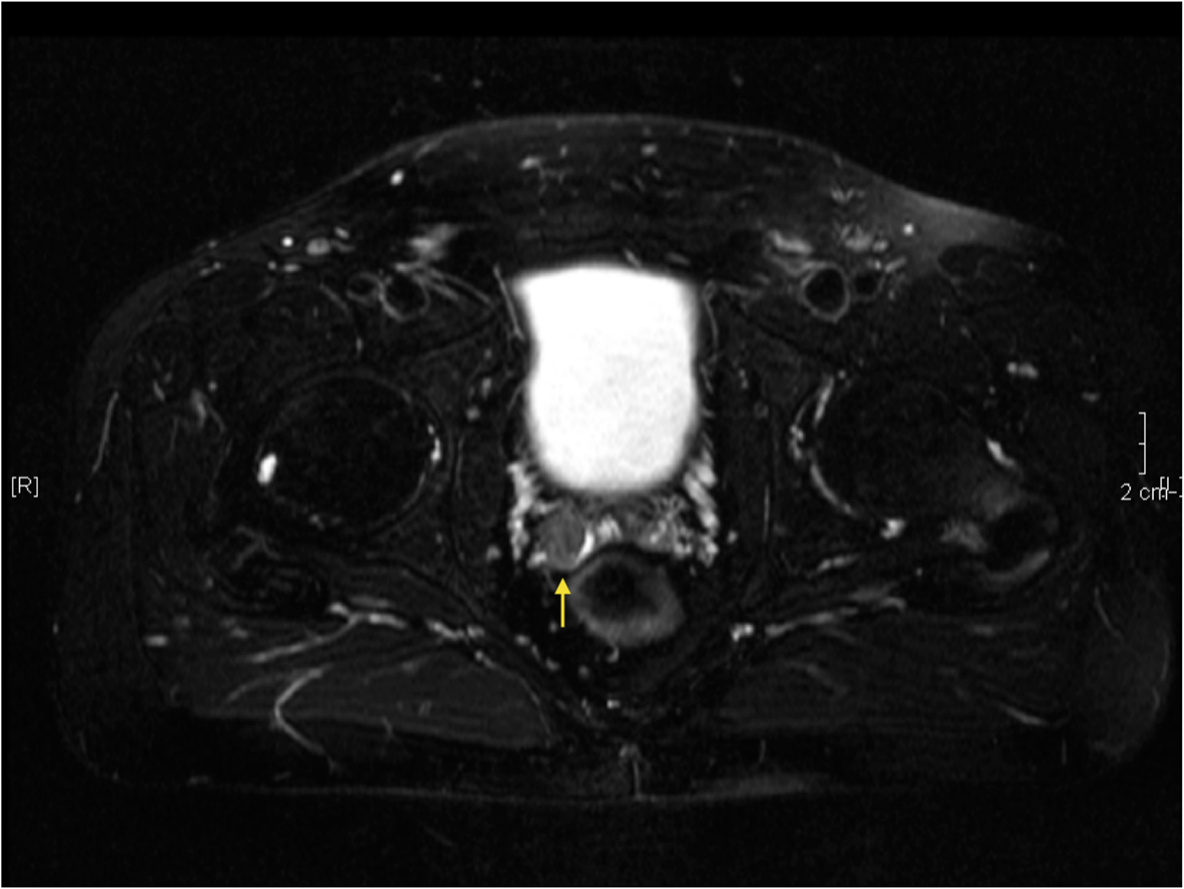
Axial T2-weighted MRI of the right seminal vesicle mass.

A forty-year old Caucasian male was referred by his general practitioner for urological assessment because of persisting, painless hemospermia, which had initially been treated with several courses of oral antibiotics for almost seven months. The patient denied prior trauma or genitourinary tract infection. The patient denied a past medical history of bleeding disorders, hypertension or malignancy of the urinary tract. On physical examination there were no signs of malignancy except a moderate swelling of the right inguinal lymph nodes. External genitalia, the prostate on digital rectal examination, the vasa deferentia, abdomen and pulmo were found to be normal. Urinalysis was sterile with a moderate microscopic hematuria of 25 Erythrocytes/μl (normally absent in urine). Full Blood Count, prostate specific antigen (PSA) level and clotting was normal. Semen analysis for the presence of melanospermia was not performed. Transrectal ultrasound (TRUS) showed a normal prostate of 35 cm^3^ and a round, hypo echoic, solid mass measuring 19 × 16 mm within the right seminal vesicle (Figure 1). The left seminal vesicle appeared normal. The lesion was further investigated by magnetic resonance imaging (MRI) of the pelvis confirming the ultrasound finding (Figure 2).

**Figure 3:**
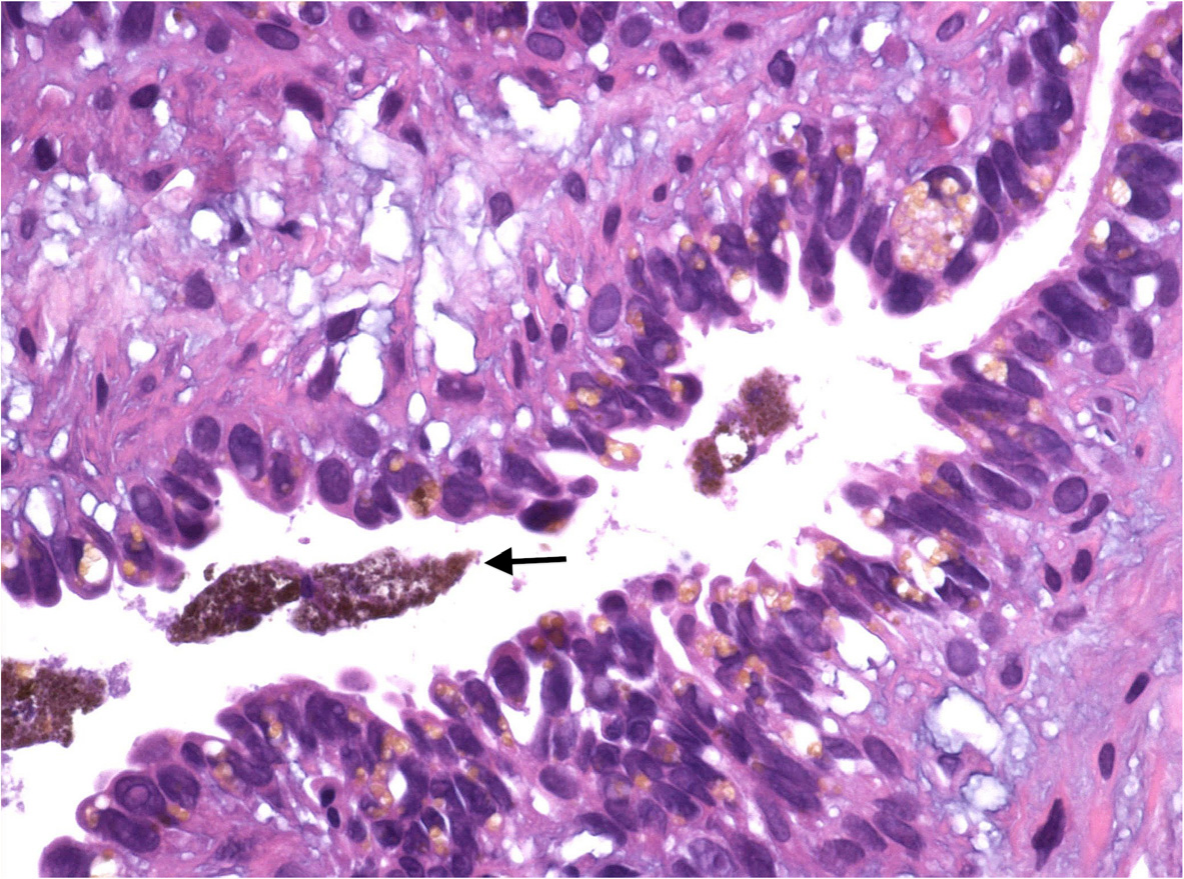
Hematoxylin-Eosin staining (x100).

**Figure 4:**
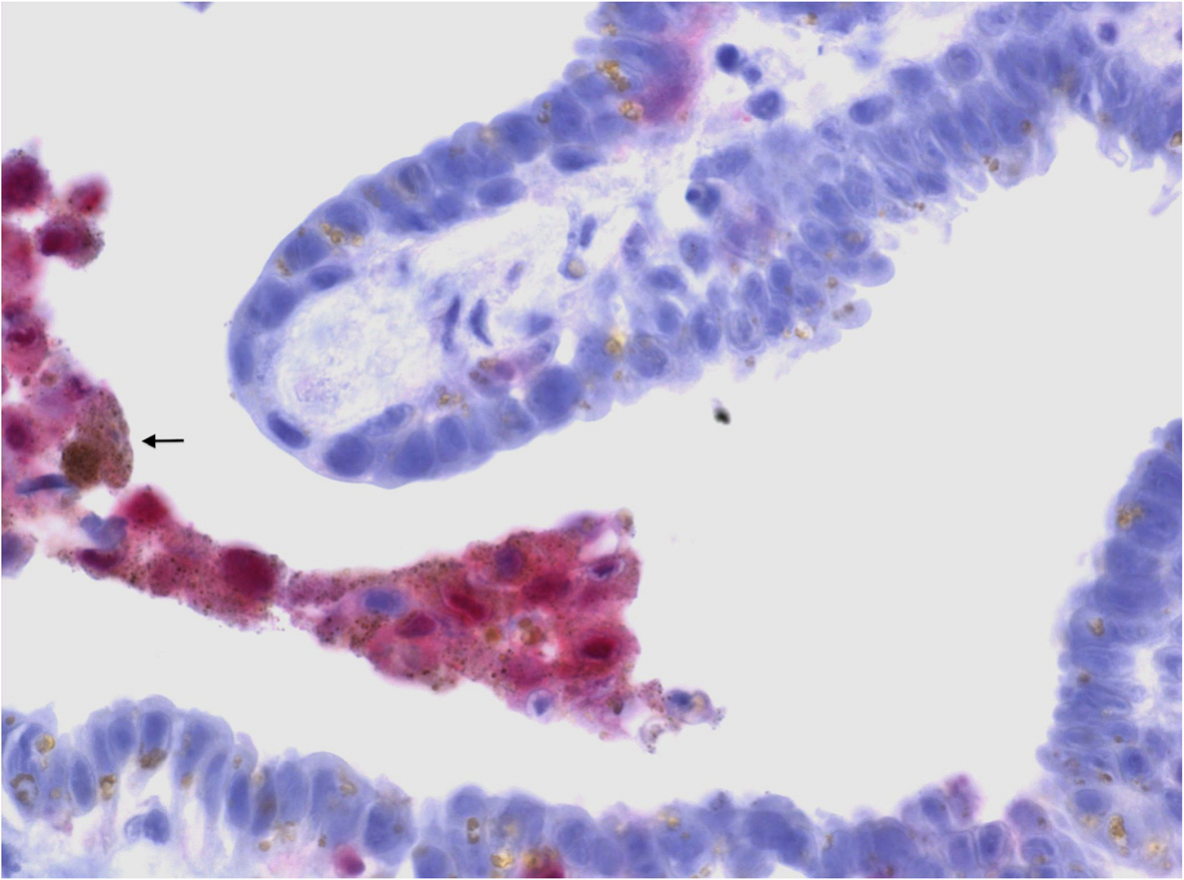
Immunostaining of the biopsy specimen with S-100 (x400).

In order to assess the nature of this suspicious lesion the patient underwent transrectal ultrasound (TRUS) guided biopsy of the right seminal vesicle. Histopathology revealed infiltration of the right seminal vesicle with strongly pigmented melanoma cells positive for S 100 and Hematoxylin-Eosin representing metastatic disease and was negative for Cytokeratines AE1/3 (Figures 3 and 4).

S 100 is a sensitive immunohistochemical marker for melanoma and belongs to the family of calcium binding proteins such as calmodulin and troponin C. It is localized in the cytoplasma and the nucleus of the cell and is divided in two subunits namely S100A and S100B. Although highly sensitive for melanomas and their metastases, is also expressed in non-melanocytic tumours such as Schwannommas, glial and neural cells and Langerhans cells.

Hematoxylin-Eosin staining on the other hand is a typical histological staining and not an immunohistochemical marker as S100. It has been in use for more than a century and is still an essential tool for recognizing various tissue types. Hematoxylin has a blue-purple color and stains nucleic acids. Eosin is pink in color and stains proteins nonspecifically. Hematoxylin-Eosin staining is also used for the measurement of tumour thickness, which is also known as the Breslow thickness, and represents a main prognostic factor for melanoma.

In our try to locate the primary site of the melanoma, dermatological assessment and computed tomography (CT) of both thorax and abdomen were performed. While pulmonary, hepatic and supraclavicular lymph node metastasis were found, the primary site of the melanoma remained unidentified. Since no primary site of the melanoma could be identified a melanoma of unknown primary was finally diagnosed.

The case entered our institutional multidisciplinary tumor board. Following respective recommendation the patient was included into an adequate clinical trial (EORTC 18032 Study) [1].

Despite active treatments in both arms the disease remained progressive. Following a further institutional multidisciplinary tumor board consensus, the supraclavicular lymph nodes and pulmonary masses were resected revealing largely necrotic metastases of the malignant melanoma. Subsequently further progression occurred and the patient refused any further specific therapy. He succumbed six months later to his progressive disease.

## Discussion

Hemospermia is defined as macroscopic presence of blood in the semen. Most cases are related to iatrogenic, inflammatory, traumatic or infectious pathologies provoking mucosal irritation, hyperemia and edema of the duct/gland, thus causing bleeding. In most of the cases hemospermia is mild and self-limited [2-4].

A detailed history eliciting issues of trauma, infection and bleeding disorders can narrow the differential diagnosis and preclude additional investigations in most patients with hemospermia. Physical examination in these patients should include blood pressure measurement, assessment of the penis, palpation of the vasa deferentia and digital rectal examination. Further radiographic workup is indicated only in an uncommon situation of palpable seminal vesicles or if a midline structure is noted on digital rectal examination.

Hemospermia secondary to malignancy most often is related to prostate cancer but even in this circumstance is rare. The incidence rate of hemospemia in male populations undergoing screening for prostate cancer has been reported to be 0,5% [5].

In cases of suspicious masses of the seminal vesicles, TRUS is the initial diagnostic tool of choice due to excellent visualization of the seminal vesicles and adjacent structures. At the same time it offers the opportunity of performing a TRUS-guided biopsy of the seminal vesicles for histological confirmation [6]. Additional radiological evaluation with magnetic resonance imaging (MRI) and computed tomography (CT) is required to visualize the exact extend of metastases and concomitant pelvic pathology [7]. Further treatment of hemospermia related to metastatic involvement strictly depends on the underlying cause but is compulsory in any case. A multidisciplinary approach focusing in further assessment and treatment options should be sought.

The CUP-Syndrom comprises a heterogenous group of metastatic tumors for which no primary site can be detected. Although most CUP-Syndromes derive from neuroendocrine carcinomas, metastatic melanomas are but sporadically detected by metastasis only [8,9].

In our case the primary malignancy has been identified as melanoma but the initial localization of the tumour remained unknown.

Only one case of metastatic melanoma of the seminal vesicles has been published to date. The previous reported case of hemospermia related to metastasis of a malignant melanoma of unidentified primary site presented with metastatic masses in both seminal vesicles with concomitant human immunodeficiency virus infection [10]. This patient presented with synchronous brain metastases subject to gamma-knife surgery and adjuvant immunotherapy with interferon. This patient died five months after the initial diagnosis. The initial location of the melanoma remained in this case also unknown.

Those two cases may suggest a need to consider hemospermia as a rare symptom of metastatic malignant melanoma to the seminal vesicles although we overall believe that most cases of hemospermia are benign in nature.

## Conclusions

Malignant melanoma should be considered as a differential diagnosis of hemospermia after common causes have been ruled out.

## Consent

Written informed consent was obtained from the patient for publication of this Case report and any accompanying images. A copy of the written consent is available for review by the Editor of this journal.

## Abbreviations

PSA: Prostatic specific antigen; TRUS: Transrectal ultrasound; MRI: Magnetic resonance imaging; CT: Computed tomography; CUP: Cancer of unknown primary.

## Competing interests

The authors declare that they have no competing interests.

## Authors’ contributions

NP was responsible for concept, design, acquisition and interpretation of data. MB contributed to design and critical revision of the manuscript and HR was responsible for the critical revision of the manuscript. All authors read and approved the final manuscript.

## Pre-publication history

The pre-publication history for this paper can be accessed here:

http://www.biomedcentral.com/1471-2490/13/43/prepub
